# Mueller matrix-based characterization of cervical tissue sections: a quantitative comparison of polar and differential decomposition methods

**DOI:** 10.1117/1.JBO.29.5.052916

**Published:** 2024-02-07

**Authors:** Nishkarsh Kumar, Jeeban Kumar Nayak, Asima Pradhan, Nirmalya Ghosh

**Affiliations:** a Indian Institue of Technology Kanpur, Department of Physics, Kanpur, Kanpur, India; b Indian Institute of Science Education and Research Kolkata, Department of Physical Sciences, Mohanpur, India; c Indian Institue of Technology Kanpur, Centre for Lasers and Photonics, Kanpur, Kanpur, India

**Keywords:** Mueller matrix, cervical tissue, tissue polarimetry, polar decomposition, differential decomposition

## Abstract

**Significance:**

Quantitative optical polarimetry has received considerable recent attention owing to its potential for being an efficient diagnosis and characterizing tool with potential applications in biomedical research and various other disciplines. In this regard, it is crucial to validate various Mueller matrix (MM) decomposition methods, which are utilized to extract and quantify the intrinsic individual polarization anisotropy properties of various complex optical media.

**Aim:**

To quantitatively compare the performance of both polar and differential MM decomposition methods for probing the structural and morphological changes in complex optical media through analyzing their intrinsic individual polarization parameters, which are extracted using the respective decomposition algorithms. We also intend to utilize the decomposition-derived anisotropy parameters to distinguish among the cervical tissues with different grades of cervical intraepithelial neoplasia (CIN) and to characterize the healing efficiency of an organic crystal.

**Approach:**

Polarization MM of the cervical tissues with different grades of CIN and the different stages of the self-healing crystal are recorded with a home-built MM imaging setup in the transmission detection geometry with a spatial resolution of ≈400  nm. The measured MMs are then processed with both the polar and differential MM decomposition methods to extract the individual polarization parameters of the respective samples. The derived polarization parameters are further analyzed to validate and compare the performance of both the MM decomposition methods for probing and characterizing the structural changes in the respective investigated optical media through their decomposition-derived intrinsic individual polarization properties.

**Results:**

Pronounced differences in the decomposed-derived polarization anisotropy parameters are observed for cervical tissue sections with different grades of CIN. While a significant increase in the depolarization parameter (Δ) is obtained with the increment of CIN stages for both the polar [Δ=0.32 for CIN grade one (CIN-I) and Δ=0.53 for CIN grade two (CIN-II))] and differential (Δ=0.35 for CIN-I and Δ=0.56 for CIN-II) decomposition methods, a trend reversal is seen for the linear diattenuation parameter $\left(d_{L}\right)$, indicating the structural distortion in the cervical morphology due to the CIN disease. More importantly, with the differential decomposition algorithm, the magnitude of the derived $d_{L}$ parameter decreases from 0.26 to 0.19 with the progression of CIN, which was not being probed by the polar decomposition method.

**Conclusion:**

Our results demonstrate that the differential decomposition of MM holds certain advantages over the polar decomposition method to characterize and probe the structural changes in the cervical tissues with different grades of CIN. Although the quantified individual polarization parameters obtained through both the MM decomposition methods can be used as useful metrics to characterize various optical media, in case of complex turbid media such as biological tissues, incorporation of the differential decomposition technique may yield more efficient information. Also, the study highlights the utilization of MM polarimetry with an appropriate decomposition technique as an efficient diagnostic and characterizing tool in the realm of biomedical clinical research, and various other disciplines.

## 1 Introduction

Many of the natural objects with biological or nonbiological origin possess some intrinsic polarization anisotropy such as birefringence, dichroism, and depolarization. Quantification of these polarization anisotropic parameters with desirable spatial resolution can provide essential morphological, structural, and functional information regarding a specimen. Hence, quantitative polarimetry has emerged as a powerful tool for characterizing a wide range of optical media in various disciplines.^1^^–^^4^ In the realm of quantitative polarimetry, polarization-resolved Mueller matrix (MM) imaging is a widely adopted technique as it can probe and quantify the complete polarization response of a sample in a single experimental embodiment.^1^^,^^2^^,^^5^^–^^7^ Owing to the recent developments in the field of MM polarimetry such as increasing the measurement precision by optimizing the calibration methods,^1^ adoption of snapshot techniques to extract the polarization information of dynamic objects,^8^ the establishment of various MM decomposition and analysis techniques to extract the polarization anisotropy parameters, etc.;^9^ the MM imaging has gained a special place in biomedical research and various other disciplines.^1^^,^^10^

However, carrying out quantitative polarimetry in optically thick turbid media such as biological tissues and other complex media is still a challenge.^2^^,^^11^^,^^12^ Although such media possess intrinsic polarization anisotropy, the inevitable depolarization through multiple scattering, and simultaneous exhibition of several polarization effects obstruct the quantification of these polarization anisotropy parameters. Therefore, in recent years, a number of MM decomposition methods were proposed aiming to extract and quantify the individual polarization anisotropy parameters in a lumped system.^1^^,^^13^^–^^15^ Spatial mapping of these decomposition-derived polarization parameters throughout a specimen contains a wealth of information regarding the medium properties. The proposed decomposition techniques come with different assumptions, and hence, one has to be judicious while choosing an appropriate decomposition technique according to the targeted real-life applications.

Some of the recent studies have shown that among the existing decomposition methods, the polar^9^^,^^16^^–^^18^ and differential matrix decomposition^14^^,^^15^ are the efficient MM decomposition techniques to extract the individual polarization parameters in complex media, exhibiting multiple polarization effects. In polar decomposition, a given MM is decomposed into sequential product of three basis matrices corresponding to the three general polarization effects [depolarization, diattenuation (linear and circular), and retardance (linear and circular)]. Yet owing to the non-commuting nature of the matrix multiplication, there is an ambiguity in the derived anisotropic parameters depending on the order of multiplication.^7^ In contrast, the differential matrix formalism is a more general kind of decomposition method, which considers simultaneous exhibition of multiple polarization effects. The mathematical description of both the MM decomposition technique is briefly discussed in the theory section.

It is crucial to validate and compare the utilization of both the polar and differential MM decomposition algorithms in case of various complex optical media, which is the primary objective of our study. To demonstrate this, we have considered two different complex optical media: (1) cervical tissue sections with varying grades of cervical intraepithelial neoplasia (CIN)^19^^–^^21^ and (2) a self-healing organic crystal at its different healing stages.^22^ Alongside being a turbid media, the cervical tissues exhibit multiple polarization effects owing to the specific orientation of the collagen fibers present within.^19^^–^^21^ More importantly, the cervical morphology is directly linked to its polarization properties, which gets modified due to the diseases such as CIN.^20^ Thus, we intend to probe the morphological changes of cervical tissues with the progression of CIN by quantifying the spatially varying polarization anisotropy parameters such as diattenuation, retardance, and depolarization. Both the polar and differential MM decomposition methods are utilized to derive these individual polarization parameters, which will facilitate quantitative comparison between these decomposition algorithms for extraction and quantification of intrinsic polarization properties in case of such complex optical media.^23^^–^^25^ Similarly, the chosen organic crystal exhibits strong polarization anisotropy effects due to its ordered crystalline structure. While subjected to external stress, the internal structural orientation gets altered and owing to its self-healing mechanism, the crystal restores its pristine property.^22^ We quantify the spatially varying polarization parameters throughout the crystal at the different stages of healing using both the polar and differential decomposition algorithms. The derived polarization parameters not only characterize the self-healing efficiency of the organic crystal but also provide a comparison between the utilized decomposition techniques.

In this work, the polarization properties of various cervical tissues and organic crystals are measured with a home built MM imaging setup in the transmission detection geometry. The recorded MMs are then processed with both polar and differential decomposition algorithms to extract and quantify the associated individual polarization anisotropy parameters. The spatial mapping of the derived individual polarization parameters is further analyzed by plotting the subsequent histograms, and the mean (μ) and standard deviation (σ) of the polarization parameters are obtained by fitting the histograms with the Gaussian distribution. The mean and standard deviations of the individual polarization parameters are then used as essential metrics to distinguish between the different grades of CIN. Our results show that, with the differential decomposition method, the obtained anisotropy parameters can efficiently distinguish the stages of CIN, which is not achieved with the polar decomposition technique. Thus, the obtained results not only enable the realization of a quantitative polarimetric diagnostic tool for the detection of CIN, but also facilitate quantitative comparison between the performances of various MM decomposition methods to characterize turbid media such as biological tissues. In addition, we have also characterized the self-healing efficiency of the organic crystal and validated the utilization of different MM decomposition techniques in such optical media.

## 2 Materials and Methods

### 2.1 Materials

Sections of 20  μm thickness from the stromal region of the cervical tissues are used in this study. A total number of 20 tissue sections are investigated in this study. Tissue sections are collected from 10 patients (two section from each patients), out of which 4 of them were suffered from CIN grade one (CIN-I), 5 were affected by CIN grade two (CIN-II), and 1 was diagnosed with CIN grade three (CIN-III). The cervical tissues are obtained from GSVM Medical College, Kanpur, and the sectioning of these cervical tissues is done by Dr. Asha Agarwal.

### 2.2 MM Polarimetry Setup

**Fig. 1:**
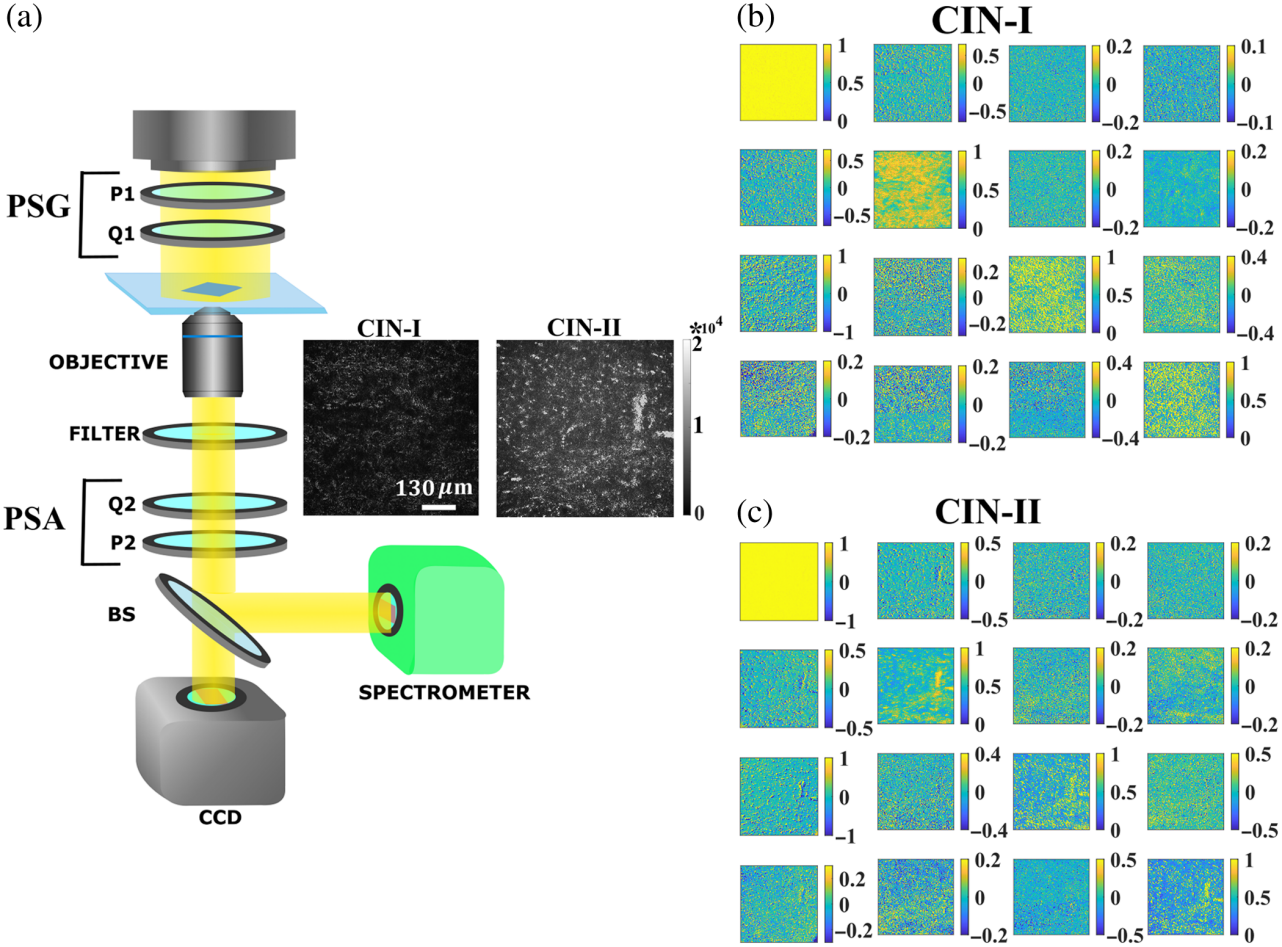
(a) Schematic of the polarization MM microscopy system to capture the spectral and imaging polarization response of a sample. PSG, polarization state generator; PSA, polarization state analyzer; P, polarizer; Q, quarter wave-plate; BS, beam splitter. The 4×4 polarization MM images of the cervical tissue section with (b) CIN-I and (c) CIN-II are presented. In addition to significant depolarization, the simultaneous occurrence of multiple polarization effects are manifested in the different MM elements. The depicted scale bar is 130  μm.

We have utilized a custom-designed polarization microscopic arrangement, where one can determine the complete polarization response of a sample by recording the 4×4 MM^22^^,^^26^^,^^27^ [Fig. 1(a)]. The experimental system employs broadband white light excitation and the subsequent recording of the polarization-resolved images of the sample at any selected wavelengths between (λ=400−725  nm). Alongside imaging, the experimental arrangement also facilitates simulated spectral MM measurements. The 36 projective polarization measurements required for the construction of the MM are recorded by sequentially generating and analyzing 6 different linear and circular polarization states.^28^^,^^29^ A collimated white light from the microscope’s (Olympus, IX-71) inbuilt illumination source (halogen lamp, JC 12V 100W) is passed through a polarization state generator unit, which comprises a rotatable (1) linear polarizer and (2) achromatic quarter wave plate (QWP, Thorlabs AQWP05M-600). Polarization of the transmitted light is subsequently analyzed by the polarization state analyzer unit that consists of a rotatable achromatic QWP and a linear polarizer. All the optical polarizing elements are mounted on computer-controlled motorized rotational mounts (PRM1/M-Z7E, Thorlabs, United States) for precision control. The MM images at a specific wavelength can be captured by putting a band-pass filter in the optical path and using an electron multiplying charge-coupled device (EM CCD) camera (Andor, IXON 3). The polarization-resolved intensity images of the cervical tissue sections (dimension = 650  μm×650  μm) are recorded using a 650 nm band-pass filter (Δλ≈25  nm). The MMs of the cervical tissue sections are constructed using 36 polarization-resolved projective measurements (given in Table 1).^28^

**Table 1:** Scheme for construction of MM using 36 polarization-resolved projective measurements. Here, the first letter represents the input polarization state, and the second letter stands for the analyzer or the projected polarization state. The states are defined as $I_{H}(\text{horizontal})$, $I_{V}(\text{vertical})$, $I_{P}(+45\;\deg)$, and $I_{M}(-45\;\deg)$, $I_{L}$ left circular polarized, and $I_{R}$ right circular polarized.

HH + HV + VH + VV	HH + HV – VH – VV	PH + PV – MH – MV	RH + RV – LH – LV
HH – HV + VH – VV	HH – HV – VH + VV	PH – PV – MH + MV	RH – RV – LH + LV
HP + VP – HM – VM	HP – VP – HM + VM	PP – PM – MP + MM	RP – RM – LP + LM
HR + VR – HL – VL	HR – VR – HL + VL	PR – PL – MR + ML	RR – RL – LR + LL

## 3 Theory

Here, we briefly describe the theoretical treatment for the extraction of polarization anisotropy parameters using both polar and differential MM decomposition methods.^7^^,^^11^^,^^13^ In the polar decomposition method, a given MM is decomposed into a sequential product of three basis matrices

$$M=M_{\Delta }.M_{R}.M_{D},$$ (1)

where $M_{\Delta }$ corresponds to the depolarization effect associated with the medium, and $M_{R}$ and $M_{D}$ describe the retardance (linear and circular) and diattenuation (linear and circular) effects, respectively. The magnitudes of the anisotropy parameters, diattenuation (D), retardance (δ), and depolarization (Δ) are calculated using the respective basis matrices.

In contrast, in the differential matrix formalism, the anisotropic polarization and depolarization effects are stored simultaneously in various elements of a differential matrix m.^14^^,^^15^ The differential matrix is related to the MM M and its spatial derivative along the direction $\left(\overset{\to }{z}\right)$ of propagation of light as^30^

$$\frac{dM}{dz}=mM.$$ (2)

Equation (2) assumes that the sample is laterally homogeneous, and both polarization and depolarization effects are occurring simultaneously. In uniformly distributed polarization properties along the propagation direction, the integration of differential matrix equation yields

L=ln M=m.l, (3)

where L is the matrix logarithm of recorded MM M, and l represents the optical path length in the medium. The polarization properties of the underlying system can be constructed using the Lorentz symmetric $\left(L_{u}\right)$ and Lorentz antisymmetric $\left(L_{m}\right)$ components of logarithmic MM $L=L_{m}+L_{u}$ as

$$L_{m}=1/2(L-GL^{T}G),\;L_{u}=1/2(L+GL^{T}G),$$ (4)

where G is the Minkowski metric tensor represented as G=diag(1,−1,−1,−1). The corresponding anisotropic parameters can be calculated directly from the respective matrix elements of the $L_{m}$ matrix.

## 4 Results and Discussion

The polarization properties of the cervical tissue sections with different grades of CIN are probed and quantified by recording the corresponding MM images in the transmission detection geometry [Fig. 1(a)]. The obtained MMs for the cervical tissue sections with CIN-I and CIN-II are presented in Figs. 1(b) and 1(c), respectively. Polarization-resolved images of the cervical tissue sections are captured with a spatial resolution of ≈400  nm, and the full 4×4 MM is constructed from the projective polarization measurements as described in Table 1. From the experimentally observed MM for both CIN-I [Fig. 1(b)] and CIN-II [Fig. 1(c)] tissue sections, it is evident that the cervical tissues exhibit multiple polarization effects such as linear diattenuation $\left(d_{L}\right)$, linear retardance $\left(\delta_{L}\right)$, and depolarization (Δ). The pronounced magnitude of the polarization anisotropic effects such as linear diattenuation $\left(d_{L})(M_{12/21},M_{13/31}\right)$ and linear retardance $\left(\delta_{L})(M_{23/32},M_{24/42}\right)$ is evident from the non-zero magnitude of the respective MM elements. In addition to linear diattenuation and retardance effects, significant depolarization of the incident polarized light is also observed, which is expected owing to the multiple scattering in the turbid tissue media.

It is pertinent to note that although the intrinsic polarization properties of the cervical tissue sections are reflected in the respective MM elements, the obtained MMs [Figs. 1(b) and 1(c)] do not possess a proper symmetry as generally observed for the conventional polarizing optical elements.^29^ This deviation originates due to the presence of significant depolarization effect and simultaneous occurrence of multiple polarization effects, which are manifested in a complex inter-related way in the MM. Thus, utilization of the MM decomposition techniques is indispensable to extract and quantify the intrinsic individual polarization anisotropy parameters. The decomposition-derived polarization parameters can be analyzed to examine the performance of various MM decomposition techniques, and in this way, it enables us to prepare efficient quantitative polarimetry methodologies for the characterization of such complex optical media. Before diving into that, we very briefly discuss the origin of polarization anisotropy effects in cervical tissues.

The cervix is composed of squamous epithelium, connective tissues, and other components. Collagen is a primary component of the cervical connective tissue, and cross-linking between the individual collagen molecules leads to the formation of microfibril and collagen fibers. The structural organization of these collagen fibers links the cervical morphology to its polarization properties such as diattenuation, retardance, and depolarization. In case of CIN, the structural organization of the collagen fibers gets distorted leading to the alteration of the polarization anisotropy properties exhibited by the cervical tissues. Thus, quantitative polarization MM imaging can be an efficient diagnostic tool for precancer detection. However, as discussed earlier, carrying out quantitative polarimetry in such optical turbid media is difficult, where we have to use various MM decomposition methods to extract and quantify the associated individual polarization parameters. Interestingly, owing to their inherent properties discussed above, the cervical tissue sections can be treated as efficient platforms for the validation and comparison between the various MM decomposition algorithms, which are utilized to probe the changes in the cervical morphology through polarization parameters. For this purpose, cervical tissue sections with different grades of CIN are taken, and a quantitative comparison between the performance of polar and differential MM decomposition is carried out by characterizing their respective polarization properties.

**Fig. 2:**
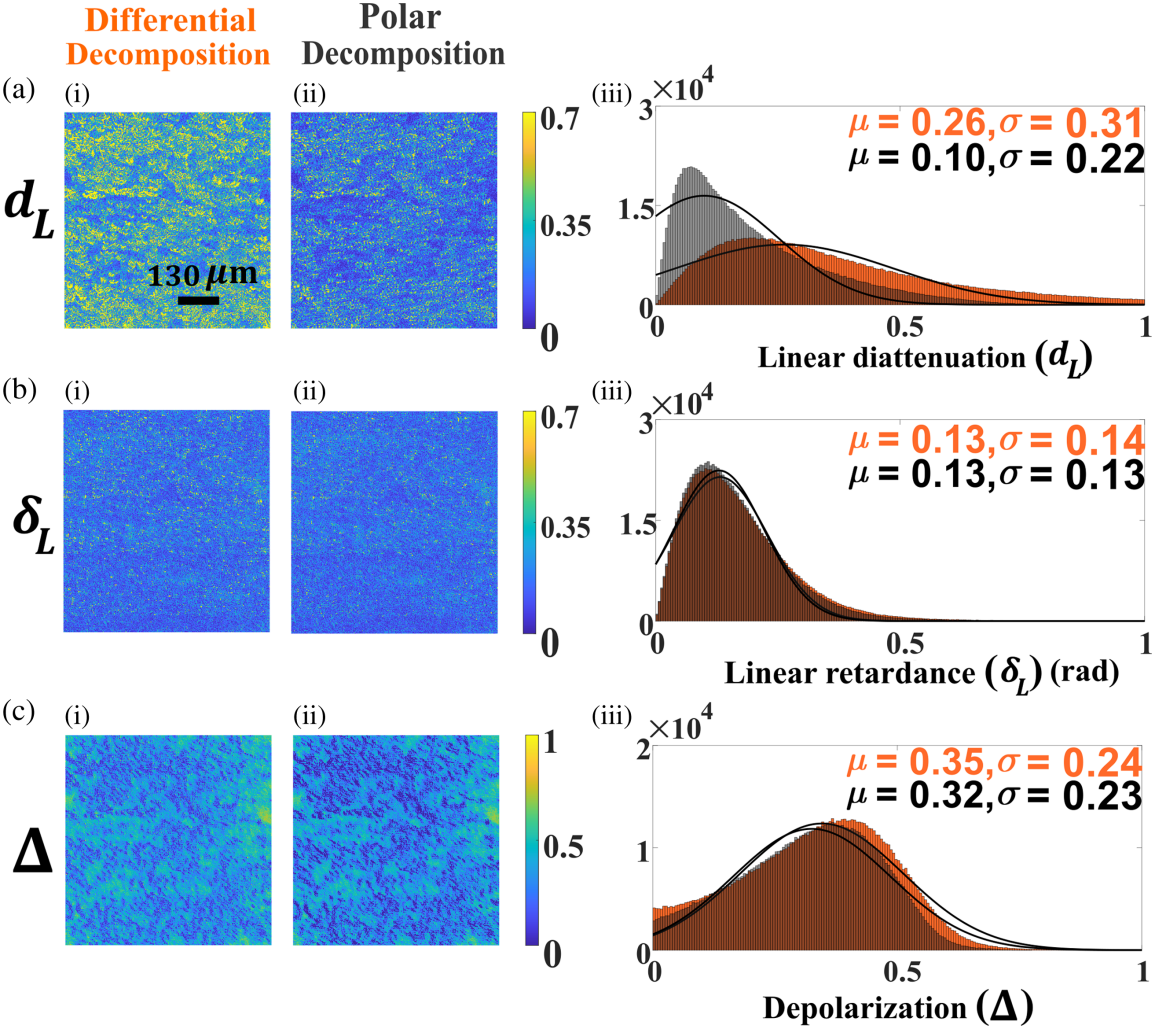
Intrinsic polarization parameters such as (a) linear diattenuation $\left(d_{L}\right)$, (b) linear retardance $\left(\delta_{L}\right)$, and (c) depolarization (Δ) are extracted and quantified from the recorded MM of the cervical tissue section with CIN-I. While the results obtained for the differential matrix decomposition method are presented in the first column [a(i), b(i), and c(i)], the spatial variation of the polarization parameters obtained with the polar decomposition method are given in the second column [a(ii), b(ii), and c(ii)]. The histogram plots corresponding to the polarization images are presented [a(iii), b(iii), and c(iii)], and further, the histograms are fitted with Gaussian distribution to calculate the statistical averages [mean (μ) and standard deviation (σ)], which are used as metrics to distinguish between the cervical tissue sections. The scale bar shown in panel [a(i)] is 130  μm.

The experimental recorded MMs [Figs. 1(b) and 1(c)] are decomposed with both polar and differential decomposition methods to extract the spatial distribution of the individual polarization parameters throughout the tissue sections, and the corresponding results are presented in Fig. 2. The associated decomposed matrices are discussed in section S1 in the Supplementary Material. Although all the derived polarization parameters such as linear diattenuation $\left(d_{L}\right)$ [Fig. 2(a)], linear retardance $\left(\delta_{L}\right)$ [Fig. 2(b)], and depolarization (Δ) [Fig. 2(c)] show significant strength, there is a spatial inhomogeneity for all the decomposition-derived polarization parameters [Figs. 2(a)(i), 2a(ii), 2(b)(i), 2(b)(ii), 2(c)(i), and 2(c)(ii)]. Hence, we go on to construct histograms for the respective polarization images, which enable a better quantitative presentation of the intrinsic polarization properties of the cervical tissue sections. Histograms shown in Figs. 2(a)(iii), 2(b)(iii), and 2(c)(iii) are plotted by taking the magnitude of the polarization parameters with number of pixels attaining that particular magnitude. Furthermore, the histograms are fitted with the Gaussian distribution, and the statistical moments of the distributions [mean (μ) and standard deviation (σ)] are calculated. Now, the mean of the individual polarization parameters can serve as more precise and convenient metrics to characterize the cervical morphology and its changes with the progression of CIN. We start with analyzing the polarization parameters associated with CIN-I cervical tissues, and compare the magnitude of the polarization parameters obtained with both the polar and differential decomposition algorithms. The magnitude of the linear retardance parameter $\left(\delta_{l}\right)$ (mean values: 0.13 and 0.13) [Fig. 2(b)(iii)] and depolarization (Δ) (mean values: 0.35 and 0.32) [Fig. 2(c)(iii)] does not show much variation between the polar and differential decomposition methods. In contrast, a significant difference in the magnitude of the linear diattenuation parameter $\left(d_{L}\right)$ is observed [Fig. 2(a)(iii)]. The magnitude of the mean values of $d_{L}$ corresponds to the differential and polar decomposition are found to be 0.26 and 0.10, respectively, which indicates the discrepancy between the polar and differential MM decomposition method.

**Fig. 3:**
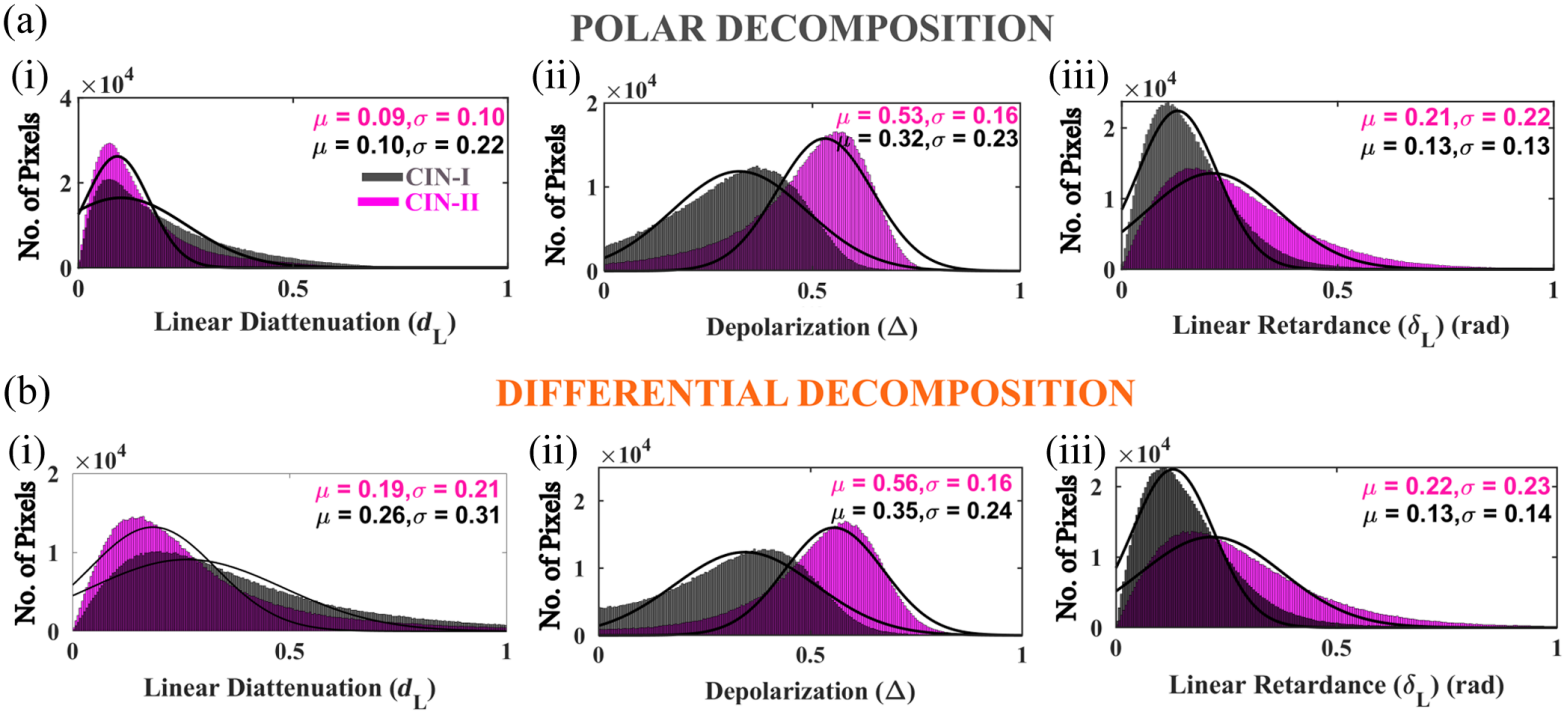
Histograms corresponding to the spatial variation of the polarization parameters derived incorporating the (a) polar and (b) differential decomposition method are presented. Comparative evaluation of both the polar and differential decomposition methods is performed by considering the mean values obtained from the Gaussian fitting. Although with polar decomposition, the linear diattenuation $\left(d_{L}\right)$ does not show much variation for CIN-I and CIN-II, a pronounced change is observed with the differential decomposition method [a(i) and b(i)]. A significant increase in the depolarization parameter with the growth of CIN is observed in the extracted polarization parameter for both polar and differential decomposition techniques [a(ii) and b(ii)]. Also increment in the magnitude of the linear retardance $\left(\delta_{L}\right)$ parameter is observed with the progression of CIN stages [a(iii) and b(iii)].

Next, we go on to quantify the associated polarization parameters of the cervical tissue sections with CIN-II following the similar methodologies as it was for CIN-I. Both the polar and differential MM decomposition methods are utilized to derive the spatial variation of the polarization parameters, and subsequent construction of histograms and their fitting with Gaussian distribution are executed to obtain the mean and standard deviation of the respective polarization parameters. The results are presented in Fig. 3 for both polar [Fig. 3(a)] and differential decomposition methods [Fig. 3(b)] so that besides demonstrating a quantitative polarimetric diagnosis tool, a detailed comparison between the performance of the polar and differential MM decomposition algorithms can be carried out. While the histograms with gray color correspond to the CIN-I cervical tissue sections, the magenta color histograms describe the spatially mapping of the polarization parameters for CIN-II cervical tissue sections. With the polar decomposition method, the mean value of the linear diattenuation parameter $\left(d_{L}\right)$ obtained for both CIN-I (μ=0.10) and CIN-II (μ=0.09) is nearly equal [Fig. 3(a)(i)]. However, a pronounced difference in the depolarization (Δ) [Fig. 3(a)(ii)] and linear retardance $\left(\delta_{L}\right)$ [Fig. 3(a)(iii)] parameters between the CIN-I and CIN-II is observed. The mean value of the Δ increases for CIN-II (Δ=0.53) as compared to the CIN-I (Δ=0.32). The mean values of the linear retardance parameter $\left(\delta_{L}\right)$ also exhibit some changes, where the CIN-II $\left(\delta_{L}=0.21\right)$ has a relatively higher magnitude with respect to the CIN-I $\left(\delta_{L}=0.13\right)$ tissue. Coming to the results obtained with the differential decomposition method, there is a pronounced change in the linear diattenuation parameter $\left(d_{L}\right)$ between the CIN-I and CIN-II tissues [Fig. 3(b)(i)]. The obtained mean value of $d_{L}$ decreases with the progress of CIN stages, and the obtained mean values for CIN-I and CIN-II are 0.26 and 0.19, respectively. This describes the degradation of the structural orientation or organization of the anisotropic collagen fibers. The results obtained for depolarization also validate this, where significant growth in the depolarization magnitude is observed in the CIN-II (Δ=0.56) as compared to the CIN-I (Δ=0.35) tissue sections [Fig. 3(b)(ii)]. However, the magnitude of the linear retardance parameter $\left(\delta_{L}\right)$ shows an increment [Fig. 3(b)(iii)] with the progression of CIN stages similar to the polar decomposition method.

From the obtained results, it is evident that the polarization parameters derived with the differential decomposition method can efficiently probe the morphological changes or here the distortion of the structural organization of the collagen fibers, which is not the case for the polar decomposition method. The decomposition-derived linear diattenuation parameters $\left(d_{L}\right)$ clearly demonstrate that, unlike the polar decomposition method, the differential decomposition method probes the reduction of the linear diattenuation parameter. Thus, the differential decomposition technique can be an efficient tool for distinguishing cervical tissues with different grades of CIN, and holds certain advantages over the polar decomposition method for characterizing complex optical turbid media such as biological tissues. In this regard, it is worth mentioning that the utilization of a differential decomposition algorithm in quantitative polarimetric measurement also supports the implication of a previous study,^13^ which highlights the usefulness of differential decomposition of MM in tissue such as media. We have also measured the polarization properties of cervical tissues with severe dysplasia (CIN-III), and the individual polarization parameters are quantified using both polar and differential decomposition methods. Although the obtained results for CIN-III exhibit similar behavior as observed for the case of CIN-I and CIN-II, these are not included in the article as the results are obtained for a single tissue section. Due to the unavailability of multiple tissue sections with CIN-III, we are not able to conduct the measurement on adequate no of samples that are required to validate the obtained conclusion. However, the results corresponding to the quantitative characterization of cervical tissues with CIN-III are presented in section S3 in the Supplementary Material. We also want to note that the other parameters of the Gaussian distributions such as skewness and kurtosis can also be used as useful metrics to distinguish between the cervical tissues with different grades of CIN.^31^

**Fig. 4:**
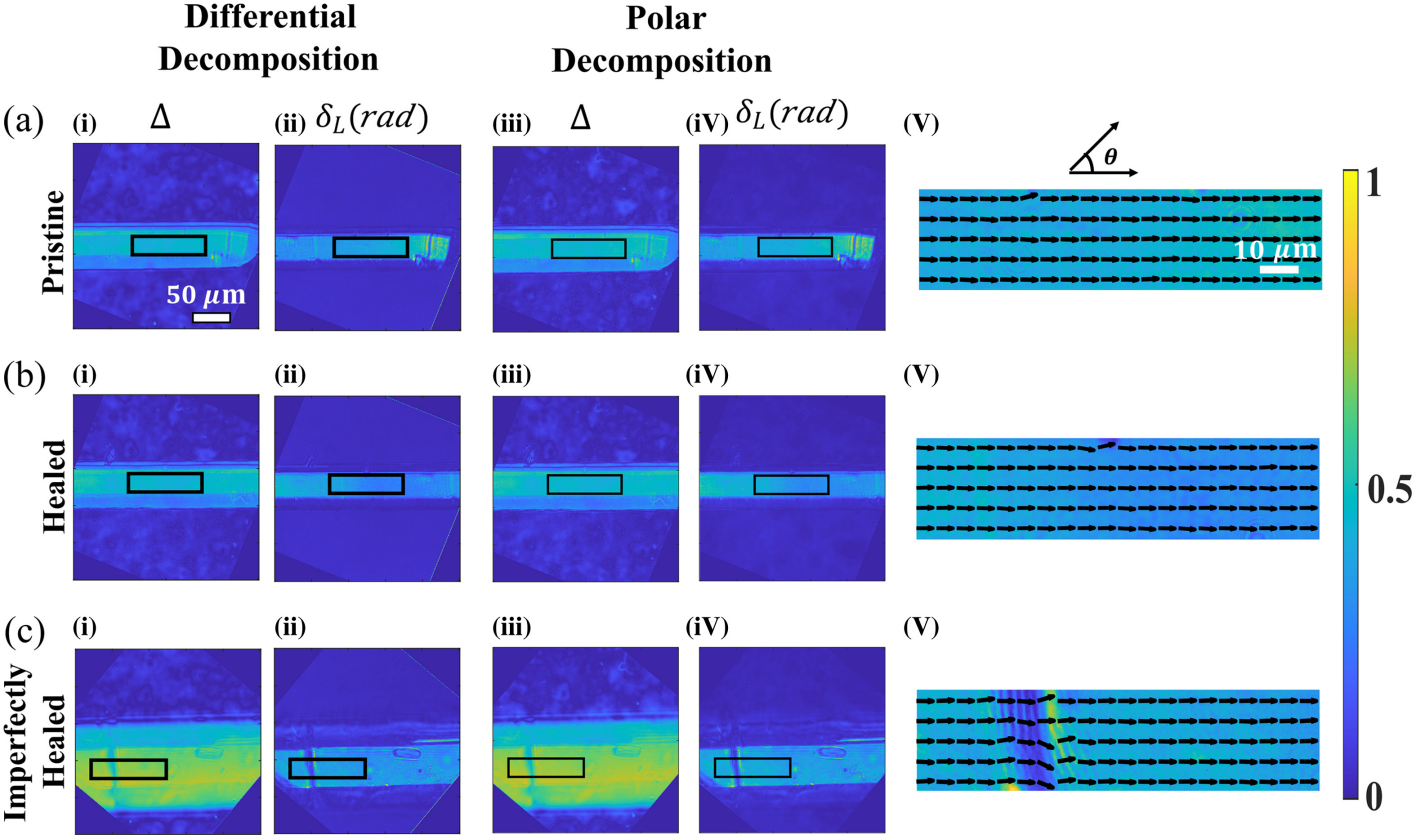
Quantified polarization parameters of the (a) pristine, (b) healed, and (c) imperfectly healed crystal are provided for both polar and differential decomposition of the MM. Spatial variations of the [(i), (ii)] depolarization (Δ) and [(iii), (iv)] linear retardance $\left(\delta_{L}\right)$ parameters are extracted from the inverse analysis of the MM. Variation in such homogeneous organic crystals is negligible while processing through different analysis methods. An area with dimensions 26  μm×104  μm (black colored box present in each image) has been analyzed for the quantitative comparison of the derived polarization parameters between the pristine, healed, and imperfectly healed crystal. (v) Spatial variation of the orientation axis of the linear retarder is also plotted for the considered region of interest (black solid box), where a clear discontinuity is visible along the crack region [c(v)] demonstrating the decrease in the linear retardance parameter. Scale bar is 50  μm.

To further compare the performance of both the polar and differential MM decomposition techniques in other complex optical media, we investigate the polarization properties of a self-healing organic crystal at its different healing stages. In our recent work, we have utilized MM polarimetry to probe the healing efficiency of a self-healing bipyrazole piezoelectric crystal.^22^ Such extraordinary crystals heal themselves anonymously when subjected to mechanical fracture through a three-point bending test. The highly ordered crystalline structure makes these crystals a polarization-rich entity, which shows strong anisotropy effects. The polarization properties at the different stages of crystals are examined in a similar way as it was shown for the cervical tissues. MM imaging of three different crystal stages such as pristine, neatly healed, and imperfectly healed is recorded, and spatial mapping of the individual polarization parameters ($d_{L}$, $\delta_{L}$ and Δ) is quantified using both the polar and differential decomposition methods. The magnitude of the decomposition-derived linear diattenuation $\left(d_{L}\right)$ parameter is found to be very small, showing a very little presence of amplitude anisotropy due to the lack of the imaginary part of the refractive index. Hence, the other two polarization parameters [linear retardance $\left(\delta_{L}\right)$ and depolarization (Δ)] are considered for the comparative study of the crystals in different stages. The corresponding results are shown in Fig. 4.

A region of 26  μm×104  μm dimension (shown with a rectangle in images of Fig. 4) is selected in each crystal through the microscope eyepiece and verified by calculating the higher correlation values for the specific dimensions between the pristine and healed crystals. Linear retardance $\left(\delta_{L}\right)$ mean values with their standard deviation for the pristine and neatly healed crystal were 0.43±0.04 and 0.34±0.05, respectively, which demonstrates the retrieval (80−85%) of the phase anisotropy of the crystal in the healing process [Figs. 4(a)(ii), 4(a)(iv), 4b(ii), and 4(b)(iv)]. The depolarization parameter Δ also shows comparable values for the pristine (0.46±0.03) and healed (0.44±0.03) crystals [Figs. 4(a)(i), 4(a)(iii), 4b(i), and 4(b)(iii)]. However, in the case of imperfectly healed crystal with clear crack junction [Fig. 4(c)], the magnitude of the depolarization parameter shows significant increase as compared to the pristine crystal [Figs. 4(c)(I),(III)]. While being under mechanical fractures, the order of the crystalline structure gets scrambled leading to the decrease in anisotropic parameters and storing their order as the crystal heals itself. Orientation angle of linear retarder axis provides a better understanding for the efficient repairing of the fractured crystal [Figs. 4(a)(v), 4(b)(v), and 4(c)(v)]. While the long-range crystalline order remains intact for both the pristine [Fig. 4(a)(v)] and the healed crystal [Fig. 4(b)(v)], a clear disorder in the orientation of the retarder axis is observed for the imperfectly healed crystal [Fig. 4(c)(v)]. The important thing to note here is that both the polar and differential decomposition of the MM give rise to the near-equal magnitude of the extracted polarization parameters, which was not the case for the cervical tissue sections. A table containing all the mean and standard deviation values of the respective polarization parameters is given in section S2 in the Supplementary Material.

## 5 Conclusion

In summary, we have presented a quantitative comparison between the polar and differential MM decomposition methods to extract and quantify the individual polarization parameters of complex optical media. Cervical tissue sections with different grades of CIN and a self-healing crystal at its different stage of healing are taken on which quantitative polarization MM measurement is carried out in the transmission geometry. The individual polarization parameters of the respective media are derived utilizing both the polar and differential decomposition algorithms. We have shown that the decomposition-derived polarization parameters can be used as essential biological metrics for distinguish between different stages of CIN. Our obtained results and consequent interpretation demonstrate that the differential decomposition of MM holds certain advantages over the polar decomposition method to probe the morphological changes in the cervical tissues with the progression of CIN. In addition, we have also investigated the performance of both polar and differential decomposition MM methods to quantify the healing efficiency of a self-healing organic crystal. Our study highlights the ability of quantitative MM polarimetry with an appropriate decomposition technique to be treated as an efficient diagnosis and characterizing tool with potential applications in various disciplines.

## Data Availability

The data and algorithms used for the MM decomposition, supporting the results reported in this study, are available from the corresponding authors upon reasonable request.
