# Supplementary material for: Mueller matrix-based characterization of cervical tissue sections: a quantitative comparison of polar and differential decomposition methods
Source: J Biomed Opt. 2024 Feb 7;29(5):052916. doi: 10.1117/1.JBO.29.5.052916 (PMC10849224; doi:10.1117/1.JBO.29.5.052916)
Supplement: Supplementary file 1 [file JBO_029_052916_SD001.pdf]

Mueller matrix based characterization of Cervical tissue sections: A quantitative comparison of Polar and Differential decomposition methods
Supplementary Information

Nishkarsh Kumar[a], **Jeeban Kumar Nayak**[b,*], **Asima Pradhan**[a,c+], **Nirmalya Ghosh**[b]
[a]Department of Physics, Indian Institue of Technology Kanpur, Kalyanpur, India- 208016.
[b]Department of Physical Sciences, Indian Institute of Science Education and Research Kolkata, Mohanpur, India-741246
[c]Center for Lasers and Photonics (CELP), Indian Institue of Technology Kanpur, Kalyanpur, India- 208016.

*Jeeban Kumar Nayak, jkn19rs027@iiserkol.ac.in +Asima Pradhan, asima@iitk.ac.in

1 S1: Differential decomposition of the imaging Mueller matrices of the cervical tissues.

As discussed in the main text, the recorded Mueller matrices of the cervical tissues are processed with both polar and differential decomposition methods to extract and quantify the individual polarization parameters. The differential decomposition of the Mueller matrix (MM) is of particular interest, as it considers the simultaneous occurrence of several polarization effects, which is the case for optical thick turbid media such as biological tissues. While briefly discussing the theoretical treatment of the differential decomposition methods in the main text, the Lorentz symmetric (L_u) and Lorentz antisymmetric (L_m) are mentioned. These two matrices $(L_m \& L_u)$ contain essential information regarding the inherent polarization properties. The off-diagonal elements of L_m yield the accumulated polarization anisotropy parameters representing the ideal scenario of the differential matrix, whereas the non-ideal polarization behaviour and the corresponding uncertainties of the accumulated anisotropy parameters are expressed in the off-diagonal elements of L_u. Thus, in the current scenario, it may be interpreted as if the polarized intensity component undergoing ideal polarization evolution is filtered into the L_m matrix by decoupling the intensity component undergoing non-ideal polarization evolution in the L_u matrix. All the accumulated sample polarization and depolarization parameters can then be quantified from the various elements of L_m and L_u. Here the resultant L_m and L_u matrices from the differential decomposition of MM are presented to describe the extraction of intrinsic polarization properties of the cervical tissues.

2 S2: Quantification of polarization parameters of an organic crystal in different stages.

The healing efficiency of a self-healing organic crystal is probed via measuring its polarization properties. Both the polar and differential decomposition methods are utilized to quantify the individual polarization parameters, which describe the polarization properties of the organic crystal in different stages. Here a table containing the mean and standard deviation values of the derived polarization parameters are provided. The results not only describe the healing efficiency but also facilitate a quantitative comparison of the MM processing with polar and differential decomposition techniques. It is observed that both the decomposition technique generates near-equal magnitude of the polarization parameters which was not the case for the cervical tissues.

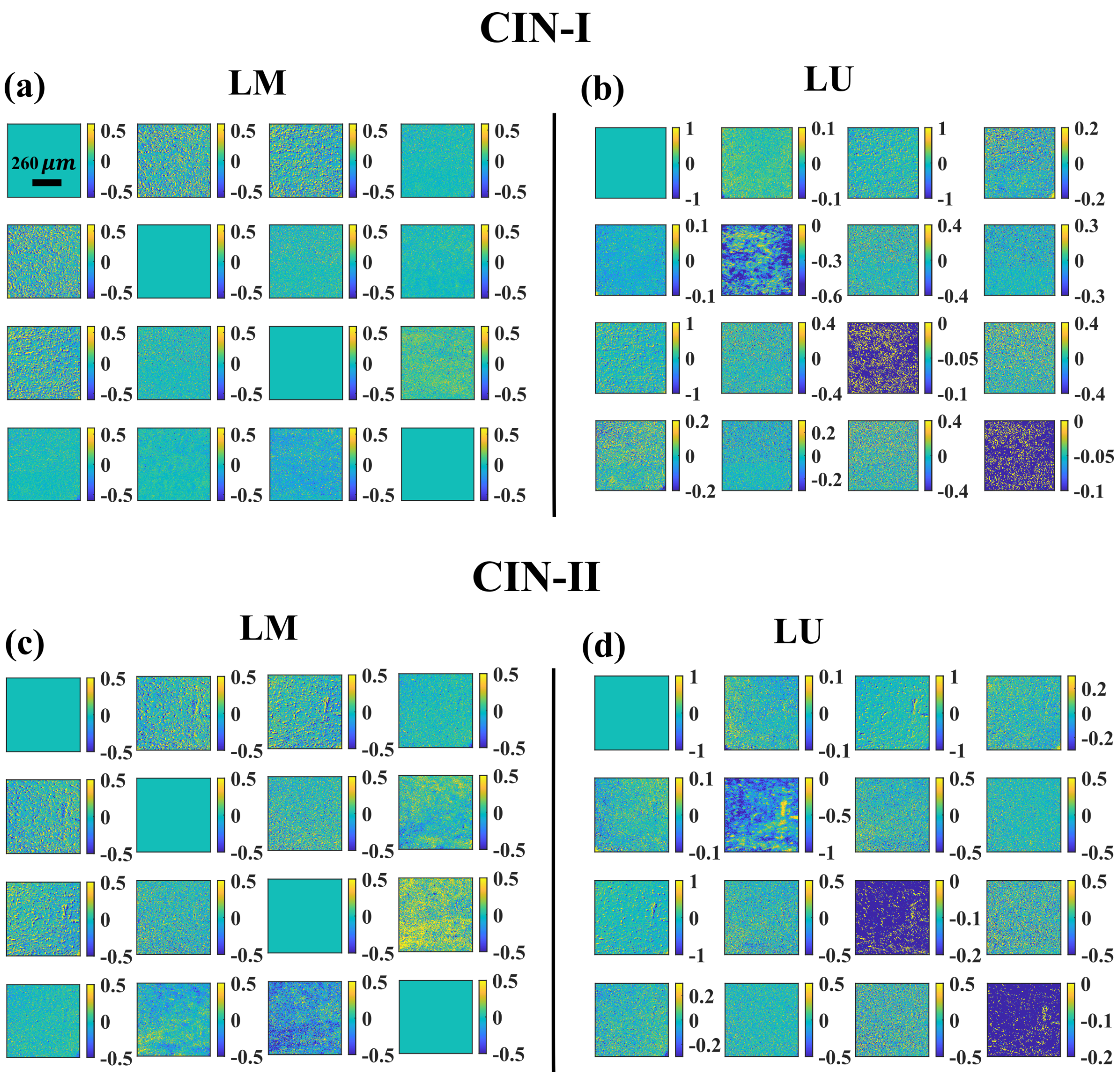

Fig 1 Lorentz anti-symmetric ((a) and (c)) and Lorentz symmetric ((b) and (d)) matrices obtained through the differential analysis method for the CIN-I and CIN-II cases.

3 S3:-Quantitative polarimetric characterization of CIN-III tissue section.

The polarization-resolved MM imaging of the cervical tissue section with CIN grade three (CIN-III) is recorded, and the individual polarization parameters are extracted/quantified by incorporating both polar and differential decomposition algorithms. The corresponding results are presented in Fig. S2. Histograms describing the spatial mapping of the decomposition-derived polarization anisotropy parameters are plotted in a similar way as given in the main text. The histograms are then fitted with the Gaussian distribution, and the obtained mean (μ) values are used as essential biological metrics to characterize the CIN grade. The linear diattenuation parameter (d_L) (0.15 obtained with differential decomposition, and 0.06 for polar decomposition)is significantly decreased in comparison to CIN-I (0.26), and CIN-II (0.19). On the other hand substantial enhancement of the depolarization parameter $(\Delta = 0.72)$ is observed with the increment of CIN grades. The

Table 1 Mean and standard deviation values of the different polarization parameters for pristine, healed and imperfectly healed crystal through polar (P.D.) and differential deomposition (D.D.) methods for the region of interest ($26\mu m \times 104\mu m$).

Parameters ↓	Pristine		Healed		Imperfectly Healed	
	P.D	D.D.	P.D.	D.D.	P.D.	D.D.
δ_L	0.43 ± 0.04	0.39 ± 0.05	0.34 ± 0.05	0.30 ± 0.05	0.38 ± 0.09	0.35 ± 0.09
d_L	0.07 ± 0.04	0.05 ± 0.03	0.08 ± 0.05	0.05 ± 0.03	0.15 ± 0.21	0.15 ± 0.21
Δ	0.46 ± 0.03	0.44 ± 0.02	0.44 ± 0.03	0.43 ± 0.02	0.68 ± 0.05	0.66 ± 0.05

observation provides strong evidence that indeed the degradation of the collagen fibers leads to a decrease in the linear diattenuation effect and an increase in the depolarization in the medium. The linear retardance parameter (δ_L) does not exhibit significant variation with respect to the value obtained for CIN-II. It is important to note that the presented results are obtained for a single tissue section due to the non-availability of cervical tissue with CIN-III, and multiple measurements on several cervical tissue sections with CIN-III are required to provide a more comprehensive result.

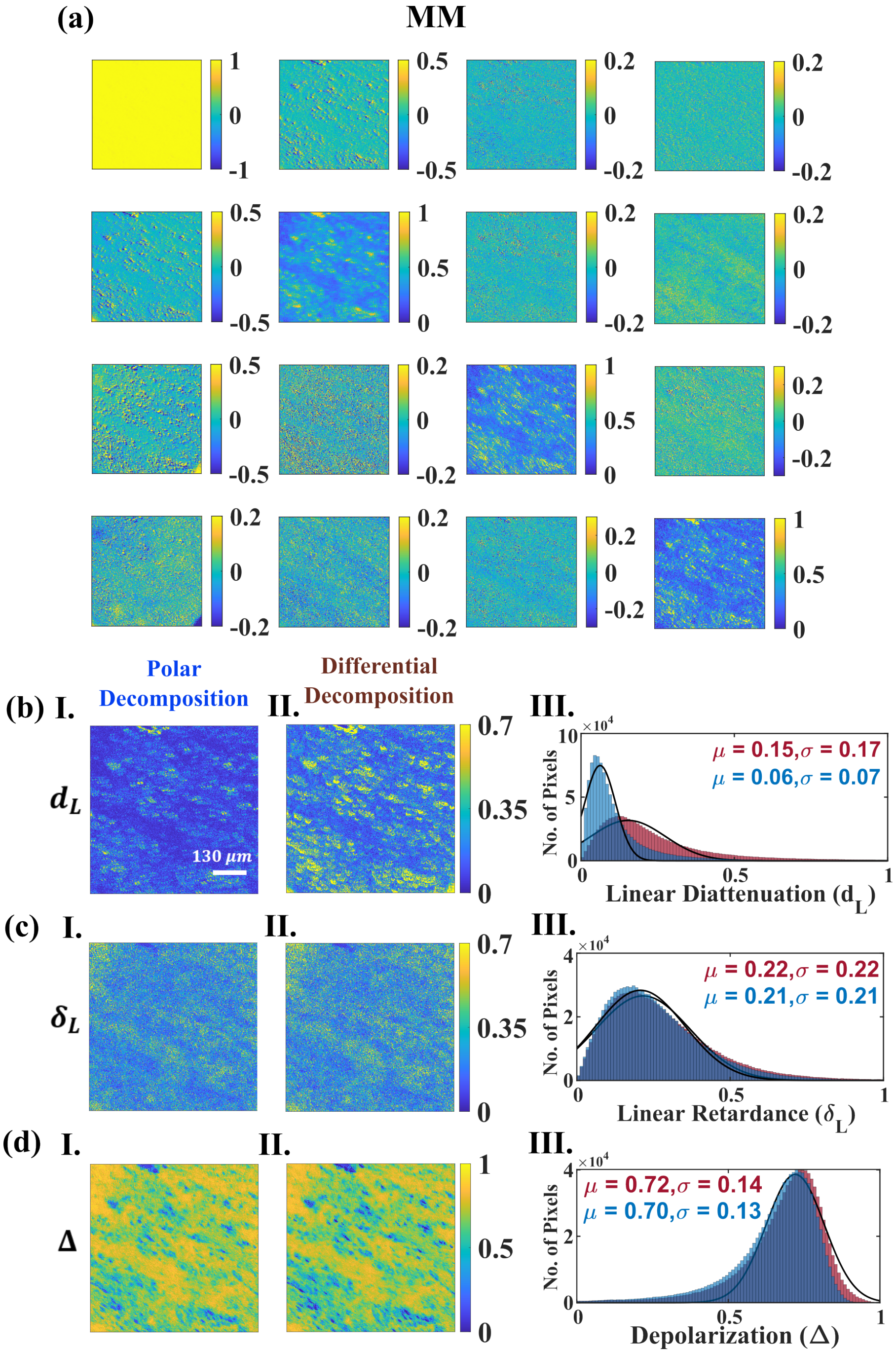

Fig 2 Quantitative polarimetric analysis of CIN-III. Polarization resolved Mueller matrix imaging of CIN-III tissue section (a). Spatial mapping of the derived intrinsic polarization parameters, linear diattenuation (d_L) (b), linear retardance (δ_L) (c), and depolarization (Δ) (d). The results obtained from both polar and differential decomposition methods are presented in the first ((b) I., (c) I., (d) I.), and second ((b) II., (c) II., (d) II.) column respectively. The histogram plots describe the spatial variation of the polarization anisotropy parameters throughout the tissue section ((b) III., (c) III., (d) III.). Blue color histograms are extracted from the polar decomposed polarization parameters while the histograms with brownish crimson color are extracted from the differentially decomposed polarization parameters.
